# Supplementary material for: Tannic Acid Induces Intestinal Dysfunction and Intestinal Microbial Dysregulation in Brandt’s Voles (Lasiopodomys brandtii)
Source: Animals (Basel). 2023 Feb 7;13(4):586. doi: 10.3390/ani13040586 (PMC9951651; doi:10.3390/ani13040586)
Supplement: Supplementary file 1 [file animals-13-00586-s001.zip › animals-2075983-supplementary materials.pdf]

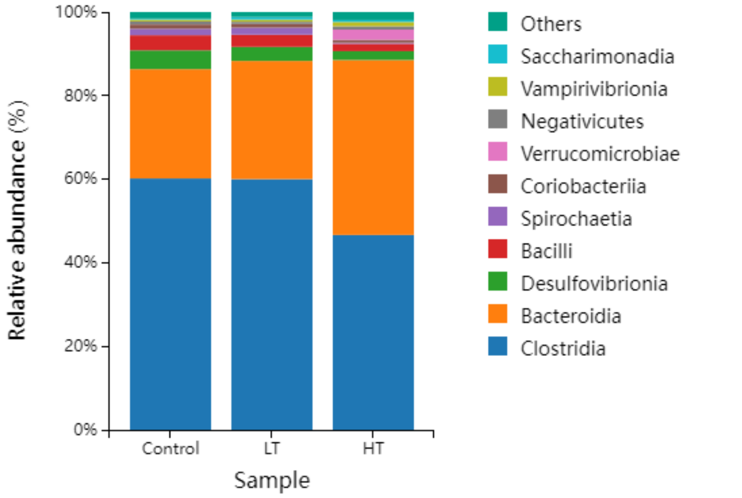

Figure S1. Relative abundance of operational taxonomic units among control group (0 mg•kg^{-1}d^{-1} TA), low TA dose group (600 mg•kg^{-1}d^{-1} TA) and high TA dose group (1200 mg•kg^{-1}d^{-1} TA) at the class level in the colonic microbiota of adult male Brandt's vole.

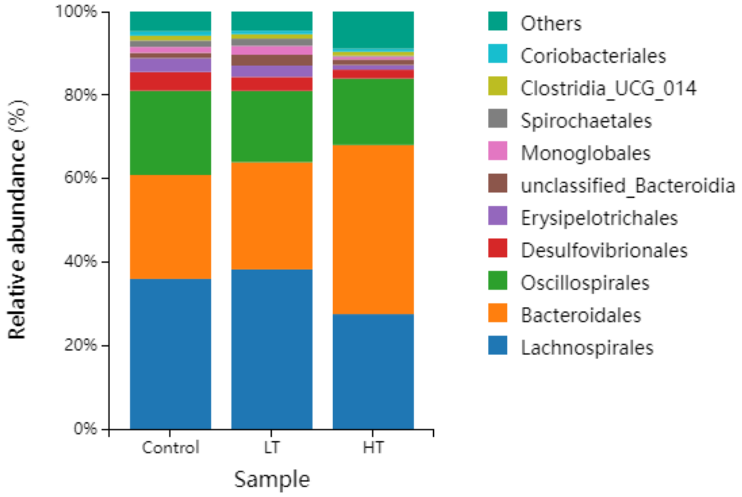

Figure S2. Relative abundance of operational taxonomic units among control group (0 mg•kg^{-1}d^{-1} TA), low TA dose group (600 mg•kg^{-1}d^{-1} TA) and high TA dose group (1200 mg•kg^{-1}d^{-1} TA) at the order level in the colonic microbiota of adult male Brandt's vole.

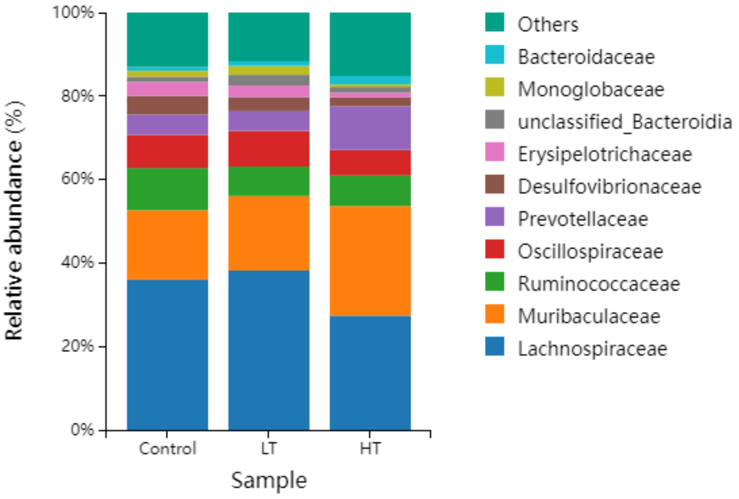

Figure S3. Relative abundance of operational taxonomic units among control group (0 mg•kg-1d-1 TA), low TA dose group (600 mg•kg-1d-1 TA) and high TA dose group (1200 mg•kg-1d-1 TA) at the families level in the colonic microbiota of adult male Brandt's vole

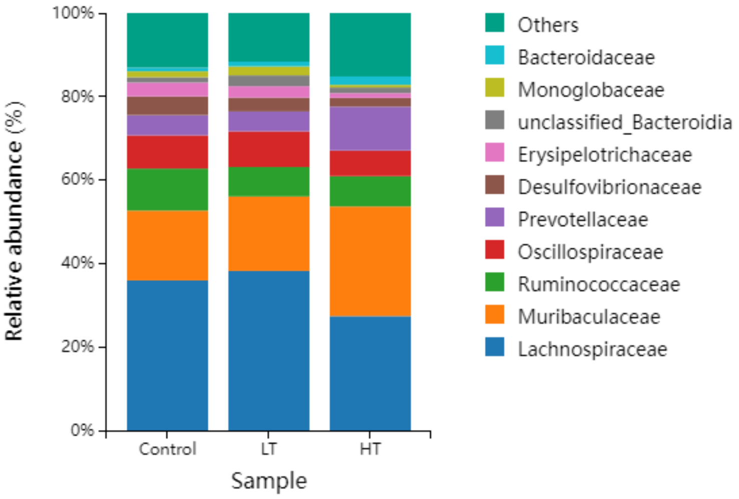

Figure S4. Relative abundance of operational taxonomic units among control group (0 mg•kg^{-1}d^{-1} TA), low TA dose group (600 mg•kg^{-1}d^{-1} TA) and high TA dose group (1200 mg•kg^{-1}d^{-1} TA) at the genus level in the colonic microbiota of adult male Brandt's vole.

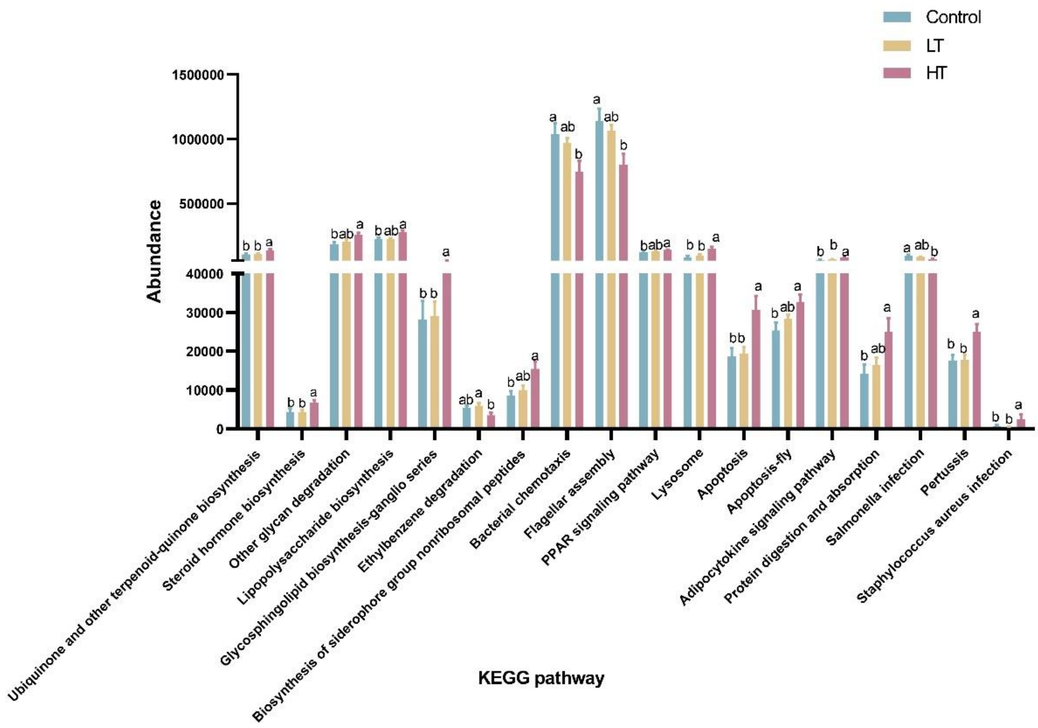

Figure S5. KEGG pathways with the significantly different abundances in the colonic microbiota of Brandt's voles. Control, 0 mg•kg^{-1}d^{-1} tannic acid; LT, 600 mg•kg^{-1}d^{-1} tannic acid; HT, 1200 mg•kg^{-1}d^{-1} tannic acid. [a, b] Means with different letters differ significantly (P < 0.05). Data are presented as mean ± SEM, n = 6.
